# An Investigation into the Determining Factors of Zoo Visitor Attendances in UK Zoos

**DOI:** 10.1371/journal.pone.0029839

**Published:** 2012-01-11

**Authors:** Andrew William Whitworth

**Affiliations:** 1 University of Glasgow; Institute of Biodiversity, Animal Health & Comparative Medicine; College of Medical, Veterinary & Life Sciences, Glasgow, United Kingdom; 2 The CREES Foundation, London, United Kingdom; University of Western Australia, Australia

## Abstract

The debate as to which animals are most beneficial to keep in zoos in terms of financial and conservative value is readily disputed; however, demographic factors have also been shown to relate to visitor numbers on an international level. The main aims of this research were: (1) To observe the distribution and location of zoos across the UK, (2) to develop a way of calculating zoo popularity in terms of the species kept within a collection and (3) to investigate the factors related to visitor numbers regarding admission costs, popularity of the collection in terms of the species kept and local demographic factors. Zoo visitor numbers were positively correlated with generated popularity ratings for zoos based on the species kept within a collection and admission prices (Pearson correlation: *n* = 34, r = 0.268, *P* = 0.126 and *n* = 34, r = −0.430, *P* = 0.011). Animal collections are aggregated around large cities and tourist regions, particularly coastal areas. No relationship between demographic variables and visitor numbers was found (Pearson correlation: *n* = 34, r = 0.268, *P* = 0.126), which suggests that the popularity of a zoo's collection relative to the types and numbers of species kept is more indicative of a collection's visitor numbers than its surrounding demographic figures. Zoos should incorporate generating high popularity scores as part of their collection planning strategies, to ensure that they thrive in the future, not only as tourist attractions but also as major conservation organizations.

## Introduction

The role of zoos has evolved over recent years [1]–[3]. It is clear that as well as contributing significantly towards biological conservation, providing an attraction for the public also commands consideration, as this is where many zoos generate the majority of their income [4].

It has been stated that the popularity of zoos has declined over the past 20 years partly as a result of a rise in competing attractions [2], [5]. Turley [2] conducted a study based on UK zoos that focused on tourism. The results indicated that the primary motivation for people visiting a zoo is pleasure, orientated towards children (75% of visiting groups contained at least one child). Not having any children was one frequently stated reason for not having visited a collection within the past three years. Turley predicts three possible futures for zoos (rejuvenation, petrification or decline) and finds petrification to be the most likely future outcome. This would mean visitors to zoos would be steady and at a lower level but would also suggest that only the most successful zoos would survive.

It has been shown by Ward et al. [6] and Ward [7] that there is a relationship between popularity and body size within zoo animals and that the loss of larger species may result in decreasing visitor numbers and adversely affect the income of the zoo. Data were collected at Zürich Zoo, Switzerland, in 1996 and assessed the popularity of 35 of the zoos exhibits. The results showed that animals of larger body size were more popular with visitors and larger species were more popular per unit cost. Balmford [8] conflicts these findings and states that there is no justification in simply keeping popular animals that in general are larger, more expensive to maintain and breed at slower rates. The case for keeping genetic rarities, in the hope that they may increase visitor numbers and resources for conservation, also comes under scrutiny within this topic of debate [9]. It is acknowledged that popular species could potentially increase the number of people visiting zoos and subsequently increase the income that could be directed towards conservation and research. Studies have attempted to identify popular zoo species but have not discovered which particular factors make species appealing [10], [11] (see Table 1).

**Table 1:** The popularity lists generated by Morris (1959).

Liked			Disliked		
1	Monkey	13%	1	Snake	28%
2	Chimpanzee	13%	2	Spider	10%
3	Horse	9%	3	Lion	5%
4	Bushbaby	8%	4	Rat	4.50%
5	Giant Panda	8%	5	Crocodile	4%
6	Bear	7%	6	Skunk	3%
7	Elephant	6%	7	Gorilla	3%
8	Lion	4.50%	8	Hippopotamus	3%
9	Dog	4%	9	Rhinoceros	3%
10	Giraffe	3%	10	Tiger	2%

Stokes [12] studied the appeal of different penguin species but focused upon finer detail features that may make one species more appealing than another. Stokes analysed 304 photographs of penguins from four different books, identified the species into eight distinct morpho-species and subjected them to analyses based upon three neotenous features (head length to body length ratio, beak length to head length ratio and relative apparent eye size) and percentage of warm colour. Factors that have previously been shown to correlate with popularity, such as the presence of neotenous features [13], [12], cultural familiarity or body size [6], [7], were not found to be the factors that made particular species of penguins more popular but warm coloration was. Through understanding the individual features that relate to popularity and positive appeal an overall level of popularity can be determined.

This type of practice in identifying charismatic species is not only essential to the zoo community but has also proved essential within a wider conservation context. Conservation organisations, zoos included [14]; focus their publicity on large charismatic species in order to raise awareness and funds [15]. Clucas et al. [15] observed the characteristics related to the flagship species found on 759 covers of ten different conservation and nature magazines in the USA. Okello et al. [16] assessed the marketing methods of ‘the big five’ charismatic large mammals in Amboseli National Park, Kenya. Evidence from the study, which involved calculating viewing times and vehicle crowding of various species, suggests that animals other than ‘the big five’ should be considered in marketing strategies.

Recent research has shown that zoo visitor attendances in the UK have actually increased within the last 20 years as opposed to decreasing, a pattern also observed in the USA [17]. Davey found a positive significant relationship between a country's population size and income in relation to zoo-attendance figures. The study focused on an international level, looking at trends between different world regions. Generally over the 40 year period Japan had the highest zoo visitor numbers, followed by North America, whilst New Zealand had the lowest, with the UK being the second lowest. Population size is an important factor determining the number of visitors to a collection on an international level but it is unclear whether details of the local population structure have an effect on visitors on national or regional levels, something that this paper addresses with regard to collections in the UK.

The aims of this investigation are listed below.

- To observe the distribution and location of zoos across the UK.
- To develop a way of calculating zoo popularity in terms of the species kept within a collection.
- To investigate the factors related to visitor numbers regarding admission costs, popularity of the collection in terms of the species kept, location and local demographic factors.

## Methods

### Ethics Statement

*Participation within this study was completely optional and only those who volunteered filled in the questionnaires. All questionnaires were kept completely anonymous. Consent was purely verbal due to the insensitive nature of the questionnaires.*

*Zoos involved either gave consent or were those zoos that provided data publicly.*

*Consent was not obtained from the University ethical committee because all data used regarding the zoos was either publicly available data or consent was delivered in the form of an e-mail from zoo management personnel. The questionnaires were non sensitive and voluntary.*

### Zoo selection

Some 55 collections distributed across the UK were included in the study but complete variable sets were available for just 34. Collections were involved in the study on the basis that they were members of British and Irish Association of Zoos and Aquariums (BIAZA) or have previously been members. The collections also had to be considered generalist collections keeping a variety of taxa.

### Demographics

The demographic data were processed using two software packages, ArcGIS and MapInfo professional v9.0. Census data was collected from CasWeb (Census area statistics on the Web) encompassing information about the total population, age structure, economic activity and household composition.

Boundary data was collected from the UKBORDERS website. The boundary data were collected for England at Wards output level. For Scotland and Wales data were collected at the Output Area level.

The boundary and the census data were joined in ArcGIS so that the required census data could be transferred to the boundary maps of England, Scotland and Wales. This permitted the three boundary data maps of England, Scotland and Wales merged together. To allow the data to be processed relatively quickly the large boundary data file was converted into point data.

The postcodes of the various zoos were used to map the location of the zoos in ArcGIS and allowed the creation of buffering layers for 15 mile (*c.* 24 km), 40 mile (*c.* 63 km) and 75 mile (*c.* 121 km) radii around each point.

The buffer and boundary data files were translated from their Environmental Systems Research Institute (ESRI) shape format into MapInfo TAB format for use in MapInfo. Fifteen miles (*c.* 24 km) was used to represent demographics on a very local scale, 40 miles (*c.* 63 km) as a mid-distance journey and 75 miles (*c.* 121 km) as close to the maximum distance to travel within a day towards a collection. These distances were chosen to account for any differences in the scale at which demographic factors may have an effect. Smaller zoos for example may rely upon immediate surrounding areas as opposed to the greater distances in which larger zoos may draw upon.

The buffer file was then overlaid onto the boundary data point file and the various demographic attributes for the total population, age structure, economic activity and household composition were calculated in each of the three distance buffer zones for each zoo.

### Mapping

The location and distribution of the collections were displayed visually using ArcGIS mapping software.

### Animal popularity

#### Pilot

A pilot questionnaire was devised that asked participants (a group of mixed MSc and BSc students *n* = 124) to list ten characteristics they found appealing and ten characteristics that they did not like about an animal. The results of this formed a list of over 100 features, which were then reduced into a smaller list of 56 features by adding those that indicated the same meaning together and removing those that were rarely stated from the list.

#### Questionnaire

The resultant list of features from the pilot survey was prepared as a questionnaire in which participants chose ‘like’, ‘dislike’ or ‘don't mind’ for each particular feature. Participants included a group of university staff and students (*n* = 84), a church group of various ages (*n* = 46) and a group of high school staff and students (*n* = 95). A general measure of animal popularity was deemed more informative than measuring popularity of animals with zoo-goers and much less problematic in terms of potentially confounding factors. Zoo-goers would potentially be a self selecting group that therefore is not representative of the population in general and would potentially be different for each zoo because visitors have been attracted by animals at the zoo they had chosen to visit. Although a measure of popularity in ‘the zoo-going public’ could have been used it would have had potential biases linked to people visiting zoos that had animals they preferred or that had featured in marketing campaigns. A general population measure is more useful as it potentially allows zoos to understand attractiveness of their collections to the public as a whole, as opposed to solely existing zoo visitors (whom individual zoos could relatively easily target themselves). The groups chosen above are comprised of a mix of genders and age groups.

#### Popularity index (devised by the authors)

For each listed feature the number of times ‘like’ was ticked (a) and the number of times ‘dislike’ was ticked (b) was summed. The number of ‘likes’ (a) and ‘dislikes’ (b) were then divided by the total number of participants (n = 225, see step 1). Finally, taking the ‘dislike’ ratio (b1) from the ‘like’ ratio (a1) generated a popularity index (c) for each characteristic (see step 2).

Step 1: Step 2:


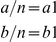
 
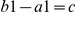


#### Applying to animal groups

A list of animal groups was developed to represent those groups kept by BIAZA members. The characteristics from the pilot questionnaire were then applied to each animal group. If a characteristic was thought to be generally representative of the animal group as a whole then it was attributed the popularity score for that particular characteristic. By summing all the attributed scores, a popularity rating was created for each animal group. This part of the process was subjective to the authors' application of characteristics to each animal group, which is why a reliability test was conducted.

#### Developing a collection score

A collection obtained the score for an animal group if they had at least one species within that particular group. A weighting was used for each additional species within the group by adding 10% of the popularity rating for that particular group. This level of 10% is arbitrary and a trial figure that added weight to those collections with greater numbers of species within one group, whilst also attempting to avoid extreme bias. The scores received for each animal group and any additional species in that group were then accumulated to provide a popularity score for each collection.

#### Reliability of assigning characteristics

In order to assess the reliability of the application of the characteristics scores to each animal group, a small sample questionnaire was designed and given to a small team of zookeepers from Trotters World of Animals, Cumbria, UK (*n* = 7). They were asked to apply the characteristics to a sample of 22 animal groups used in the study. The resultant rankings of this sample were then compared to the rankings generated by the authors as a small sample test of reliability.

### Admission costs

These figures were collected from websites or were provided by zoos over the phone (summer 2007).

### Visitor numbers

Visitor numbers were obtained from BIAZA once each zoo had granted permission for their use in the study.

### Multivariate analysis

#### Principal component analysis (PCA) and factor analysis (FA)

PCA was used on data sets containing collections with all variables present (visitor numbers, admission costs, popularity scores and demographic figures). The final data set contained 34 collections with fully complete variables.

Seven PCAs were conducted. The first encompassed popularity rating, admission costs, and the total populations within a 15, 40 and 75 mile radius (*c.* 24 km, *c.* 63 km and *c.* 121 km, respectively). Three more were conducted on the data that contained actual demographic figures whilst the final three were conducted on data containing demographic variables that consisted of proportions of the total population. Once the number of components was determined for each analysis using PCA, a FA was conducted to extract the appropriate number of factors.

## Results

### Animal popularity

The top five characteristics that were listed as ‘liked’ by the majority of the sample population were ‘active’, ‘easy to see’, ‘intelligent’, ‘bright colours’ and ‘the ability to hold objects’. The bottom five characteristics that were listed as ‘dislike’ by many of the sample population were ‘smelly’, ‘slimy’, ‘bites or stings’, ‘bald/little hair’ and ‘venomous/poisonous’ (Table 2). Characteristics that scored close to 0 (e.g. thin, sharp claws and teeth, dull coloured) represent those features that were neither likeable nor dislikeable (‘don't mind’) by the population or could be those characters that may be liked by half of the population but the positive score cancels out owing to the other half that dislike the characteristics. Some paired characters were both positive features but ‘exotic’ and ‘rare’ were more popular than ‘lives in Britain’ and ‘common’. Another observation to note is that ‘small’ scored more popular than ‘large’ when considering the size of an animal.

**Table 2:** A list of the generated characteristics from the questionnaire and the scores attributed to them from the following questionnaire.

Characteristic	Score		
Active	0.80	Feeds on plants	0.29
Easy to see	0.77	Lives alone	0.28
Intelligent	0.75	Active during night time	0.23
Bright colours	0.73	Feathers	0.18
Ability to hold objects	0.70	Slow	0.18
Furry	0.68	Unintelligent	0.16
Rare	0.68	Quiet	0.13
Active during day time	0.65	Un-patterned	0.13
Fast	0.64	Fat	0.09
Exotic	0.64	Ugly/unusual looking	0.09
Climbing	0.60	Secretive	0.06
Swimming	0.57	Thin	0.05
Big eyes	0.56	Sharp claws and teeth	0.00
Patterned	0.56	Dull coloured	−0.04
Small (smaller than a man)	0.54	Feeds on other animals	−0.12
Lives mainly on ground	0.48	Inactive	−0.14
Strong/powerful	0.46	Scaly	−0.18
Tail	0.44	Dangerous to humans	−0.24
Flying	0.40	More than 4 legs	−0.29
Lives in groups	0.38	No legs	−0.30
Frequently vocal	0.37	Aggressive to each other	−0.36
Large ears	0.35	Venomous/poisonous	−0.38
Lives mainly in trees	0.32	Bald/little hair	−0.41
Quick/erratic movements	0.32	Bites or stings	−0.45
Large (larger than a man)	0.32	Slimy	−0.52
Common	0.29	Smelly	−0.68
Lives in Britain	0.29		

Table 3 shows that primates were the most popular group of mammals, whilst ‘hippos’, ‘aardvarks’, ‘cavy-like rodents’ and ‘insectivores’ scored relatively low. ‘Passerines’ and ‘parrots’ were the two highest scoring bird groups whilst the ‘frogmouths and nightjars’ ranked lowest. With regard to the reptiles and amphibians the ‘iguanas’ and ‘frogs and toads’ were the two highest scoring, with ‘crocodiles and alligators’, ‘caecilians’ and the venomous snake groups scoring the lowest.

**Table 3:** A list of the animal groups and the scores generated once the characteristic scores had been applied to each group.

Animal group	Score	10%			
*Reptiles and amphibians:*			*Mammals:*		
Iguanas and relatives	8.25	0.82	Lesser apes	10.73	1.07
Frogs and toads	7.81	0.78	Prosimians	10.31	1.03
Tortoises and turtles	6.93	0.69	Monkeys	10.13	1.01
Anguimorph lizards	6.51	0.65	Great apes	10.04	1.00
Skinks and relatives	5.71	0.57	Squirrel-like rodents	9.44	0.94
Boas, pythons and relatives	5.69	0.57	Zebras	7.92	0.79
Geckos and snake-lizards	5.42	0.54	Elephants	7.76	0.78
Newts and salamanders	5.36	0.54	Giraffe and okapi	7.74	0.77
Colubrids	5.32	0.53	Marsupials	7.39	0.74
Tuataras	4.19	0.42	Tree shrews	7.25	0.73
Vipers	4.16	0.42	Hyraxes	7.22	0.72
Elapids	4.10	0.41	Dogs and relatives	6.84	0.68
Crocodiles and alligators	3.98	0.40	Big cats	6.72	0.67
Caecillians	3.08	0.31	Hyenas and aardwolf	6.56	0.66
			Deer	6.52	0.65
*Birds:*			Horses and asses	6.44	0.64
Parrots	9.74	0.97	Seals and sea lions	6.43	0.64
Passerines	9.14	0.91	Cattle and relatives	6.36	0.64
Kingfishers and relatives	8.33	0.83	Bears	6.35	0.63
Pigeons	7.73	0.77	Elephant shrews	6.31	0.63
Cuckoos and turacos	7.70	0.77	Raccoons and relatives	6.11	0.61
Penguins	7.65	0.77	Mustelids	5.84	0.58
Woodpeckers and toucans	7.55	0.75	Rabbits, hares and pikas	5.63	0.56
Gamebirds	7.41	0.74	Camels and relatives	5.36	0.54
Waterfowl	6.98	0.70	Mouslike rodents	5.19	0.52
Mousebirds	6.80	0.68	Civets and relatives	5.04	0.50
Birds of prey	6.59	0.66	Rhinoceroses	4.91	0.49
Trogons	6.41	0.64	Pigs	4.70	0.47
Flamingos	6.24	0.62	Small cats	4.42	0.44
Pelicans and relatives	6.18	0.62	Tapirs	4.33	0.43
Owls	6.14	0.61	Bats	3.95	0.39
Ostrich, rheas, cassowaries and emus	5.85	0.59	Monotremes	3.87	0.39
Cranes and relatives	5.66	0.57	Anteaters and relatives	3.40	0.34
Waders, gulls and auks	5.56	0.56	Insectivores	3.23	0.32
Herons and relatives	5.52	0.55	Aardvark	2.93	0.29
Nightjars and frogmouths	4.73	0.47	Cavylike rodents	2.32	0.23
			Hippopotamuses	2.17	0.22

### Zoo popularity

The zoos that scored as the top five collections based upon the popularity of their collections were Chester, Paignton, London, Edinburgh and Twycross. The five lowest scoring collections were New Forest Wildlife Conservation Park, Highland Wildlife Park, Knowsley, Shaldon and The Living Rainforest (Table 4). Despite the fact that visitor number figures cannot be stated (for confidentiality reasons), it may be noted that there are some unexpected results within the rankings. Markedly the following collections scored relatively low (*popularity scores shown below in brackets*) in comparison with their visitor number figures: Knowsley (111.36), Flamingo land (298.46), Woburn (235.39) and West Midlands (200.99).

**Table 4:** The final list of popularity scorings (for all collections that had 2006 animal inventory data available).

Zoo name	Score		
Chester	551.73	Newquay	260.43
Paignton	521.76	Thrigby	253.96
London	505.77	Africa alive	251.25
Edinburgh(c)	427.22	Curraghs(b)	249.26
Twycross	420.67	Woburn	235.39
Colchester	413.98	Camperdown(a)	225.20
Belfast(b)	404.20	Shepreth	221.62
Blackpool	399.91	Birmingham(a)	217.13
Marwell(a)	399.17	Tropiquaria	210.28
Whipsnade	379.79	West midlands	200.99
Dudley	362.29	Fota(b)	200.50
Banham	356.54	Linton(a)	198.30
Bristol(a)	352.96	Galloway(a)	189.96
Dublin(b)	322.91	Blair Drummond	174.37
Exmoor(a)	309.44	Tilgate	172.14
Paradise WLP	303.63	Tropical world(a)	165.60
Cricket St Thomas	302.75	Battersea	163.07
Jersey(b)	299.54	Lakeland	154.95
Drayton manor	299.09	Wildwood trust(a)	146.37
Flamingo Land	298.46	Calderglen	140.07
Amazon world(a)	296.02	Living Rainforest	112.16
Welsh mountain	280.22	Shaldon	111.36
Drusillas(a)	272.36	Knowsley	111.36
Trotters	269.87	HWP(c)	89.70
Chessington	264.60	New forest WCP	75.99
Birdworld	261.96		

(a) – Represents those collections that were excluded from the multivariate analysis because they either would not allow visitor number figures to be released or they did not provide permission within the timeframe needed for completion of the analysis.

(b) – Represents those collections that were excluded from the multivariate analysis because demographic figures could not be gathered from the census data used in the study.

(c) – Only a combined visitor number figure was available for Edinburgh and Highland Wildlife Park. The decision was made to only use Edinburgh in the multivariate analysis using this combined visitor number figure and the popularity rating calculated solely for Edinburgh.

### Reliability of assigning characteristics

 There was a significant positive relationship found between the ranks of the sample list given to the keeping staff (*n* = 7) and the rankings generated by the authors (Spearman's rank correlation: *n* = 22 r_s_ = 0.591 *P* = 0.01). The use of Spearman's rank was used here as n = 7 and being non-parametric was more applicable on a sample of this size.

### Multivariate analysis

Demographic variables are linked together but with slight variations with the loadings. The admission costs for adults and children were grouped together with loadings that are almost identical. There is also a correlation with admission costs and the popularity scorings of the zoos.

### Data containing actual demographic figures

The first PCA looking at popularity scores, admission costs and total populations compresses the six variables into three. The first factor of the initial FA was loaded mostly with the total populations and was accountable for 39.3% of the variation. The second factor was loaded mostly with admission prices and a minor contribution from the popularity rating and was responsible for 34.2% of the variation. A third factor was composed mostly of the popularity rating but was responsible for just 16% of the variation. The communalities for each variable all lay above 82%, which suggests they are all well represented with the three factors. The first factor may be termed ‘total population’, the second can be termed ‘zoo success’ whilst the third may be termed ‘zoo popularity’.

The above result was almost identical for each of the PCAs and FAs for the data that included demographics of age structure, household composition and economic activity. Each one resulted in just three factors being extracted with all demographic factors being loaded onto the first factor, whilst admission costs are the main loadings on the second factor, with a minor contribution from the popularity scoring. The third factor, which only accounts for a small amount of the data variation, is mainly composed of the popularity scoring for the zoos. The main difference being that over 80% of the variation in the data was retained within the first factor (‘demographics’).

### Data containing proportions of the total population

 The PCAs determined that five factors would best represent the data as >90% of the variation would be retained. Each of the following FAs had five factors extracted with a varimax rotation.

 At the 15 and 40 mile (*c.* 24 km and *c.* 63 km) distances four of the factors were loaded mainly with demographic variables. However, it was also observed that one factor was comprised mostly of admission costs and the popularity scoring on the same factor.

The 75 mile (*c.* 121 km) distance displayed the first three factors containing the various demographic variables. The fourth factor was loaded with admission costs, with a minor contribution from the popularity scoring. The final factor, which only accounts for a small amount of the data variation, was mainly composed of the popularity scoring for the zoos.

### Factor comparisons with visitor numbers

**Figure 1:**
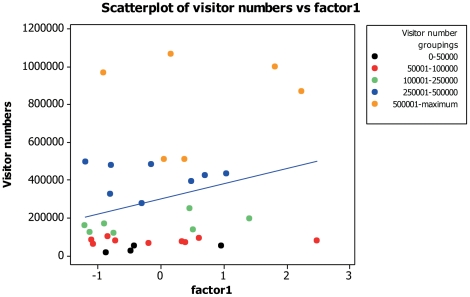
Scatter plot to display visitor numbers against residual scores of factor one (‘total populations’) from the FA.

**Figure 2:**
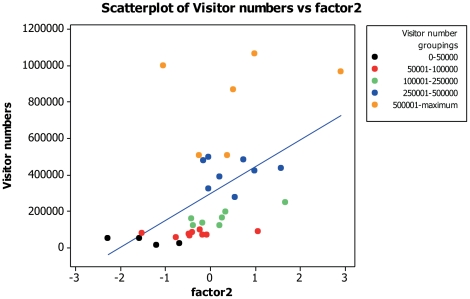
Scatter plot to display visitor numbers against residual scores of factor two (‘zoo success’) from the FA.

**Figure 3:**
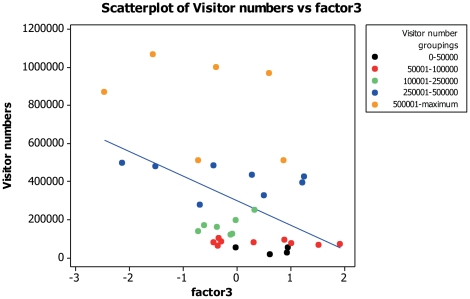
Scatter plot to display visitor numbers against residual scores of factor three (‘zoo popularity’) from the FA.

Visitor numbers displayed a positive correlation with both admission costs (for adults and children) and also with the generated popularity scores. There was no relationship to suggest that demographic factors related to a zoo's visitor numbers. When visitor numbers were compared with the first factor from the first FA no correlation was found (Pearson correlation: *n* = 34, r = 0.268, *P* = 0.126; Fig. 1). Visitor numbers were positively correlated with both factors two (Pearson correlation: *n* = 34, r = 0.496, *P* = 0.003; Fig. 2) and three (Pearson correlation: *n* = 34, r = −0.430, *P* = 0.011; Fig. 3). Pearson's correlation is used here as n = 34 and a parametric analysis is applicable.

### Demographics

**Figure 4:**
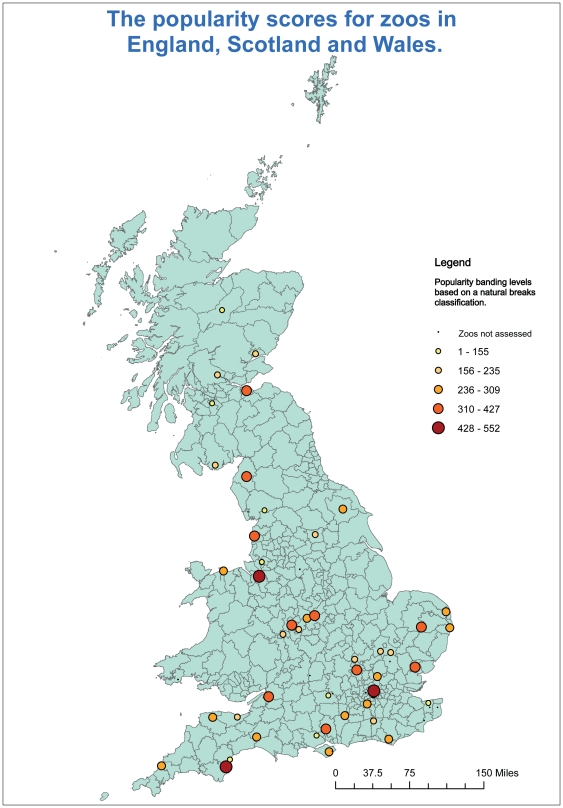
The distribution of zoos across the UK and the relative popularity scores.

The distribution of the zoos is clustered around larger cities with higher populations (e.g. London, Birmingham and the northwest) or towards areas of high tourism, such as the southwest, coastal areas and the Lake District (Fig. 4). There are differences between zoos situated in coastal regions and those situated within city areas in relation to the demographic factors within their local areas.

### Age structure

 The maps relating to age structure show that the younger age groupings are relatively widely distributed amongst all zoos. The zoos with higher proportions of 30–44 year old age groupings tend to be found within larger cities whilst zoos with higher proportions of older age groupings tend to be found around coastal areas but are very low within major cities.

### Economic activity

The zoos that have a higher proportion of economically active people within their buffer zones can be found in and around London, whilst economic inactivity and unemployment levels are much higher around zoos located in coastal areas and the northwest of England.

### Household composition

Zoos within inner city areas have a larger proportion of single-parent households whilst zoos with higher proportions of couples with children are situated surrounding city areas. Zoos in coastal areas have higher proportions of couples without children.

## Discussion

The collections located around large cities have high populations, high proportions of families or single parents with children and high proportions of economically active people. These relate to the higher proportions of 30–44 year old adults that will be of a working age close to cities. Other collections, particularly those situated in nearby coastal areas, have lower proportions of children, higher proportions of pensioners and higher levels of unemployment and economic inactivity. These locations are known tourist areas and so the collections found here rely upon tourism. A zoo's target audience has previously been stated to be the family market, which relates to high levels of parents with children within the surrounding area [2]. This would explain why city areas are popular owing to the high levels of families residing there and also coastal and tourism areas where many families choose to visit on holiday.

The characteristics scores proved very interesting in relation to previous studies regarding body size being related to the popularity of zoo animals [6]–[8]. ‘Small’ body size was shown to be more popular than ‘large’, which suggests that animals that may have previously been thought popular due to their size may not actually be regarded as charismatic for this sole reason. ‘Easy to see’ came out as the second most popular feature and would certainly have some influence on the popularity of many larger bodied species, such as elephants. The list of animal groups and their scores is a valuable way of determining which types of animals are regarded as the most popular but due to the applied characteristics there is now an empirical insight as to what makes them charismatic. The most and least popular species showed some congruence with the list generated by Morris [10], in particular the popularity of primates, elephants and giraffes and also in the dislike of snakes, crocodiles and hippopotamus.

The final rankings of the collections based upon the popularity score was close to an expected list when considering visitor numbers. The exceptions in the results (Knowsley, Flamingo Land, Woburn and West Midlands) show that there may be more complexity in developing a popularity index than simply looking at the species kept. Knowsley, Woburn and West Midlands are all safari parks and differ from the other zoos in that they provide larger, open paddocks for the majority of their animals but contain fewer numbers of species relative to their size. This indicates that the type of collection with reference to the style of enclosures/environments that the animals are kept in should be considered when calculating popularity in future. If this were the case then the safari park collections would likely have higher popularity scores. Flamingo Land is another exceptional circumstance in that it also comprises a large theme park associated with the zoo that boosts the visitor numbers disproportionately in relation to the popularity of the species kept. There may be a large influence from other facilities and amusements that zoos have to offer within their parks and should certainly be considered in future work regarding collection popularity.

The PCA and the FA analyses conducted on the data with the true figures showed a basic grouping of all demographic variables together on one factor and all the other variables that might show some indication of a zoo's success on the following two factors. The analyses conducted on the demographic data displayed as proportions of the total population show some more intricate structure to the demographic variables and load them over a greater number of factors. What is significant is that all the variables related to a ‘zoo's success’ were still loaded together onto the same factor. It appears that popularity score and the admission costs show a close relationship, so that a higher popularity scoring indicates higher admission costs.

The only significant relationships found between visitor numbers and any of the factors generated from the multivariate analyses are between the factors comprised of the popularity ratings and admission costs. The fact that none of the factors containing demographic variables showed any relationship with visitor number figures suggests that the popularity of a zoo's collection based upon the types and number of species kept is more indicative of a collection's visitor numbers than its surrounding demographics. Although it is advantageous for zoos to generate as high a popularity score as possible, it is vital that they retain other objectives and considerations with regards to species conservation activities and collection planning. Zoos with a higher popularity rating and visitor numbers can charge higher admission costs to relate to the increased popularity of the collection as a visitor attraction. Despite this finding, it is clear that many other factors clearly have a huge influence on visitor numbers, such as enclosure design, welfare standards, marketing strategies, zoo amenities and gastronomy, etc., which should be considered in future attempts to measure zoo popularity and not solely the popularity of the species within the zoos. Marketing information is commercially sensitive and was unobtainable for inclusion in this study. Not having this data does not invalidate these results however, which are general enough to be detectable even in the absence of specific marketing data (this was also a positive reason for measuring popularity in the general population rather than the zoo visitors, because it would not have been be clear if preferences were the result of an individual zoos' recent marketing strategies).

Despite the fact that populations on an international scale have been shown to relate to zoo visitor attendance figures [17], the results from this study indicate that on a finer demographic scale the popularity of the collection based upon the species kept is most significant. It would also be useful to look at collections in different continents and distinguish whether there are differences in the factors that affect visitor numbers within collections in different countries or continents at regional levels.

 I suggest that keeping species that have higher popularity scorings and also keeping a varied collection can achieve higher visitor number figures. Zoos in a healthier financial situation hold the potential to maintain high welfare standards, produce a high quality education facility and a high conservation output, three of the most important modern day aspects in zoos justifying their existence. Zoos should incorporate popularity information into their general decision making but not make it their sole focus and use this tool to help support other key activities, including the consideration of keeping species of high conservation value. If zoos use information such as this solely to guide their decision making and collection planning then they could easily lose reputation as a conservation organisation. However, it is undoubtedly essential that zoos retain popularity in order to bring in revenue and are able to aid potential future output towards conservation; a healthy balance of considerations should be met. Without this popularity consideration they could fail in their conservation objectives. Zoos can apply these methods in a combination of collection planning techniques to ensure that they thrive in the future, not only as tourist attractions but also as major conservation organizations [2], [5].
